# Subtypes in pancreatic ductal adenocarcinoma based on niche factor dependency show distinct drug treatment responses

**DOI:** 10.1186/s13046-022-02301-9

**Published:** 2022-03-10

**Authors:** Tomohiko Shinkawa, Kenoki Ohuchida, Yuki Mochida, Kukiko Sakihama, Chika Iwamoto, Toshiya Abe, Noboru Ideno, Yusuke Mizuuchi, Koji Shindo, Naoki Ikenaga, Taiki Moriyama, Kohei Nakata, Yoshinao Oda, Masafumi Nakamura

**Affiliations:** 1 Department of Surgery and Oncology, Graduate School of Medical Sciences, Kyushu University, 3-1-1 Maidashi, Fukuoka, 812-8582 Japan; 2 Department of Advanced Medical Initiatives, Graduate School of Medical Sciences, Kyushu University, Fukuoka, Japan; 3 Department of Anatomical Pathology, Graduate School of Medical Sciences, Kyushu University, Fukuoka, Japan

**Keywords:** Cancer-associated fibroblast, Tumor differentiation, Subtype-based therapy, Stroma-targeting therapy, Tumor microenvironment, Organoid, Statin

## Abstract

**Background:**

Pancreatic ductal adenocarcinoma (PDAC) is characterized by abundant stroma in which microenvironmental (niche) factors promote PDAC progression. In mouse models, reduction of the stroma increased the proportion of poorly differentiated PDAC with a worse prognosis. Here, we aimed to clarify the effects of stroma on PDAC that may define the PDAC phenotype and induce distinct therapeutic responses.

**Methods:**

The molecular features of PDAC based on differentiation grade were clarified by genome and transcriptome analysis using PDAC organoids (PDOs). We identified the dependency on niche factors that might regulate the differentiation grade. A three-dimensional co-culture model with cancer-associated fibroblasts (CAFs) was generated to determine whether CAFs provide niche factors essential for differentiated PDAC. PDOs were subtyped based on niche factor dependency, and the therapeutic responses for each subtype were compared.

**Results:**

The expression profiles of PDOs differed depending on the differentiation grade. Consistent with the distinct profiles, well differentiated types showed high niche dependency, while poorly differentiated types showed low niche dependency. The three-dimensional co-culture model revealed that well differentiated PDOs were strongly dependent on CAFs for growth, and moderately differentiated PDOs showed plasticity to change morphology depending on CAFs. Differentiated PDOs upregulated the expression of mevalonate pathway-related genes correlated with the niche dependency and were more sensitive to simvastatin than poorly differentiated PDOs.

**Conclusions:**

Our findings suggest that CAFs maintain the differentiated PDAC phenotype through secreting niche factors and induce distinct drug responses. These results may lead to the development of novel subtype-based therapeutic strategies.

**Supplementary Information:**

The online version contains supplementary material available at 10.1186/s13046-022-02301-9.

## Background

Pancreatic ductal adenocarcinoma (PDAC) is one of the most lethal cancers, with a 5-year survival rate of 9% [1]. The poor survival rate has improved only modestly over the years because of the lack of effective chemotherapeutic strategies for PDAC [2]. The current standard chemotherapy for PDAC is combination regimens such as FOLFIRINOX [3] and gemcitabine plus nab-paclitaxel [4], and the selection of these therapies is often based on patient performance status and comorbidities [5]. However, the response of individual patients to treatment is variable. Consequently, the therapeutic strategies for PDAC are lacking and remain far behind those in other solid tumors such as lung and breast cancer, in which biomarker selection for targeted therapies has dramatically improved treatment approaches and patient prognosis [6–8]. Therefore, the development of novel modalities and identification of targeted therapies for individual patients with PDAC is critical.

PDAC has been reported to increase in malignancy through interactions with the abundant stroma [9, 10]. Accordingly, reduction of the stroma could be a promising therapeutic strategy for PDAC treatment [11, 12]. However, stromal-targeting therapies have failed in clinical trials [13], and PDAC mouse models showed that depletion of stromal cancer-associated fibroblasts (CAFs) leads to more aggressive PDAC behavior through an increase in the proportion of poorly differentiated type of PDAC [14, 15], These findings indicate the need to elucidate how the cancer stroma regulates PDAC phenotypes.

Histological evaluation remains one of the gold standards for the clinical characterization of tumors, and the histological grading for PDAC involves a three-tiered system based on tumor differentiation [16]. Recent RNA expression analyses of several PDAC cohorts using bulk tumor samples identified two major transcriptional subtypes with distinct prognoses and biological features. The “Classical” or progenitor subtype was characterized by the expression of epithelial markers and favorable prognosis, whereas the “Basal-like,” squamous, or quasi-mesenchymal subtype was characterized by the expression of mesenchymal markers and aggressive clinical behavior [17–19]. Although these molecular subtypes of PDAC may theoretically provide new insights for precision medicine approaches, there is still no consensus on the practical application of the subtype classification for clinical decision-making in PDAC [20]. In addition, the number of available preclinical models that reflect these subtypes is limited. For example, all conventional two-dimensional pancreatic cancer cell lines (PCCs) are classified as the “Basal-like” subtype [17]. Therefore, developing subtype-based therapeutic strategies remains challenging [21].

PDAC organoid (PDO) models can be established from an individual patient’s tumors with high efficiency and recapitulate their biological and morphological features in three-dimensional (3D) culture [22, 23]. Accordingly, the established PDOs of various phenotypes may demonstrate the biological diversity of PDAC and differences in drug response depending on the phenotype. Furthermore, co-culture models of PDOs with stromal cells such as CAFs can accurately recapitulate actual PDAC tissues, allowing evaluation of the differences in cancer stromal interactions among PDO phenotypes. Therefore, investigation of the relationship between PDAC phenotypes and cancer stroma using PDO models may enable the development of novel subtype-based therapeutic strategies.

In this study, we established PDOs and clarified their molecular features based on differentiation grade. Well differentiated PDOs showed high dependency on microenvironmental factors derived from CAFs, whereas poorly differentiated PDOs showed low dependency. Moreover, subtype classification based on microenvironmental factor dependency revealed that each subtype showed distinct drug responses. These results suggest that CAFs maintain the differentiated PDAC phenotype with a better prognosis via secretion of microenvironmental factors, further inducing differences in drug response. Our findings may provide new insights into the development of stromal-targeted therapy and subtype-based therapeutic strategies.

## Methods

### Human PDAC tissue samples

All tumor samples used in this study were obtained from patients who underwent surgery for PDAC at Kyushu University Hospital (Fukuoka, Japan); all patients provided written informed consent. Clinical data including histopathological findings were obtained from electronic medical records. The study was approved by the Ethics Committee of Kyushu University (approval number: 29–401, 30–230, 2019–462, and 858–00) and conducted according to the Ethical Guidelines for Human Genome/Gene Research enacted by the Japanese Government and Helsinki Declaration.

### Human PDAC organoids

All PDOs were established from human PDAC tissue samples as previously described [22, 24]. PDAC tissues were washed vigorously with ice-cold phosphate-buffered saline (PBS) and minced into 1 mm^3^ fragments with scalpels. The fragments were digested into single cells using the Tumor Dissociation Kit (130–095-929; Miltenyi Biotec, San Diego, CA, USA). The dissociated cells were cultured in a 24-well plate (353,504; Corning, NY, USA) with growth factor–reduced Matrigel (356,231; Corning) and cultured in niche medium (see below) at 37 °C in a humidified atmosphere containing 10% CO_2_. The culture media were exchanged every 2 or 3 days; for passaging, established PDOs were collected, washed with 0.5 mM EDTA-PBS, and dissociated by digestion with trypsin and mechanical shearing. The dissociated cells of PDO were replated with fresh Matrigel and cultured in niche medium. The following four types of media were used depending on the experiment. The basic medium for PDO culture comprised Advanced Dulbecco’s modified Eagle’s medium (DMEM)/F12 (12,634,010; ThermoFisher, Waltham, MA, USA) supplemented with 10 mM HEPES (ThermoFisher), 2 mM GlutaMax (35,050–061; Life Technologies, Carlsbad, CA, USA), penicillin/streptomycin (15,140,122; ThermoFisher), 1X B27 (17,504,044; ThermoFisher), 10 mM nicotinamide (N0636; Sigma-Aldrich Co., St. Louis, MO, USA), and 1 mM N-acetyl-L-cysteine (A9165; Sigma-Aldrich Co.). The niche medium comprised the basic medium supplemented with the following niche factors: 100 ng/ml human recombinant Wnt-3a (5036-WN-010; R&D Systems, Minneapolis, MN, USA), 1 μg/ml human recombinant R-spondin1 (RSPO1, 120–38; PeproTech, Cranbury, NJ, USA), 100 ng/ml human recombinant Noggin (120-10C; PeproTech), 50 ng/ml human recombinant EGF (AF-100–15; PeproTech), 100 ng/ml human recombinant FGF-10 (100–26; PeproTech), and A83-01 (2939/10; R&D Systems). The serum medium comprised the basic medium supplemented with only 5% fetal bovine serum (FBS). The combined medium comprised the basic medium supplemented with both niche factors and 5% FBS. All media were supplemented with Y-27263 (Sigma-Aldrich, St. Louis, MO, USA) to prevent anoikis [25, 26]. PDO images were acquired by fluorescence microscopy (BZ-X700; Keyence, Osaka, Japan).

### Human CAFs and PCCs

Two human CAF lines (CAF-1 and CAF-2) were established in our laboratory from fresh pancreatic cancer surgical specimens using the outgrowth method [9] as previously described [10, 11]. The isolated cells were confirmed as CAFs by their spindle-shaped morphology and expression of α-smooth muscle actin (αSMA) and glial fibrillary acidic protein (GFAP); immortalization of CAFs was conducted as previously described [12]. Six PCCs were used in this study: MIAPaCa-2 (Japanese Cancer Resource Bank, Osaka, Japan), Panc-1 (RIKEN BRC, Tsukuba, Japan), Capan-2, CFPAC-1, BxPC-3 (American Type Culture Collection, VA, USA), and KP-2 (Japan Health Sciences Foundation, Tokyo, Japan). All PCCs were regularly authenticated by matched short tandem repeat DNA profiling. CAFs and PCCs were maintained in DMEM (Sigma Chemical Co., St. Louis, MO, USA) supplemented with 10% FBS, streptomycin (100 mg/ml), and penicillin (100 mg/ml) at 37 °C in a humidified atmosphere containing 10% CO_2_ [27].

### Gene mutation analysis

DNA was isolated using a QIAamp DNA Mini Kit (Qiagen, Hamburg, Germany) according to the manufacturer’s instructions. The quality of DNA specimens was confirmed by gel electrophoresis. DNA samples of PDOs were amplified using the Ion AmpliSeq Comprehensive Cancer Panel (ThermoFisher). Amplified fragments were used for library preparation with Ion AmpliSeq Library Kit 2.0 (ThermoFisher) and sequence analysis by Ion Torrent Personal Genome Machine (ThermoFisher). The sequence reads were checked and mapped against the human reference sequence Hg19 by Ion Reporter Software (ThermoFisher). Torrent_variant_caller (http://158.129.170.67/ion-docs/Home.html) was used for detection for the variants. SnpEff 4.1 [28] was used for single nucleotide polymorphism annotation.

### Transcriptome analysis

Total RNA was extracted from PDOs that had been passaged 4 to 6 rounds post-establishment and cultured with combined medium at day 5 post-passage using the High Pure RNA Isolation kit (11,828,665,001; Roche, Basel, Switzerland) with DNase I (Roche). RNA quality was evaluated using 2200 TapeStaton (Agilent Technology, Santa Clara, CA, USA). Gene expression levels were determined by SurePrint G3 Human GE Microarray 8 × 60 K v3.0 (Agilent Technology). Relative hybridization intensities and background hybridization values were calculated using Feature Extraction software (Agilent Technology). The raw signal intensities of all samples were log2-transformed and normalized by quantile algorithm with the ‘preprocessCore’ library package [29] on Bioconductor software [30]. Principal component analysis (PCA) for clustering of PDOs were performed on all genes dataset using R program with rgl package. The gene expression heatmap was generated with gplots and ggplot2 package on R (https://CRAN.R-project.org/package=gplots) and MultiExperiment Viewer version 4.9. For the validation study of Moffitt’s classification, the expression data of “Basal-like” and “Classical” signature genes were extracted and normalized to a Z-score. Total score was calculated by subtracting the total Z-score of “Classical” genes from the total Z-score of “Basal-like” genes; PDOs with total score ≥ 0 were classified as “Basal-like” subtype, and PDOs with total score < 0 as “Classical” subtype. Gene set enrichment analysis (GSEA) was performed for two clusters identified by PCA using GSEA software version 4.0.3 (Broad Institute, UC San Diego, CA, USA). For the analysis of genes with expression levels that were positively or negatively correlated with niche dependency scores, the Pearson correlation coefficients of the basal dataset on R software were calculated and the top 1000 and the bottom 1000 correlated genes were extracted. Functional annotation clustering analysis was performed using DAVID (National Institute of Allergy and Infectious Diseases).

### Proliferation and organoid formation assays

For proliferation assay, the growth rate of PDOs and 3D-cultured cell lines was evaluated using the CellTiter-Glo Luminescent Cell Viability Assay Kit (CellTiter-Glo Kit, G7571; Promega, Madison, WI, USA). Cells (5,000) were seeded into a 24-well plate with Matrigel and cultured under indicated conditions. To measure the amount of cells in each well, the 3D-cultured cells were trypsinized into single cells again for each well. The cells for each well were then replaced into the 96-well plates (655,083; Greiner Bio-One International, Kremsünster, Austria) with CellTiter-Glo reagents and the luminescence was measured by a microplate reader (Infinite200, TECAN, Männedorf, Switzerland) according to the manufacturer’s instructions. A blank well containing only medium was used to define baseline luminescence of the medium, and the luminescent signal for each sample was calculated by subtracting the baseline luminescence from the luminescence of each well. In assays to compare the proliferation of PDOs in different medium, the luminescent signal of 5,000 cells were measured as a control on day 0, and the proliferation fold change in luminescent signal on day 10 relative to the control was calculated. For organoid formation assay, phase-contrast images of PDOs were captured using BZ-X700 with the Z-stack and image stitching function on day 10. Fully focused images were generated and the number and total area of PDOs for each well was quantified using the HybridCellCount software module of BZ-X Analyzer. An area of 2000 μm^2^ and more was identified as an organoid. Niche dependency scores were calculating by the ratio of the proliferation fold change in niche medium to that in serum medium. In niche dependency assays, PDOs were cultured in niche medium or in niche medium lacking indicated factors and the growth rate was evaluated on day 10. The proliferation fold change in niche medium lacking the indicated factors relative to niche medium was calculated. In C59 assays, PDOs were cultured in niche medium lacking Wnt3A (-Wnt medium) supplemented with vehicle (dimethyl sulfoxide, DMSO) or in -Wnt medium supplemented with 100 nM porcupine inhibitor (Porcn-i; C59, ab142216; abcam) and the growth rate was evaluated on day 10. In RSPO1 vs. RSPO3 assays, Grade1 PDOs were cultured in niche medium with the indicated concentration of RSPO1 or RSPO3 (recombinant human RSPO3, 120–44; PeproTech) and the growth rate was evaluated on day 8. The proliferation fold change of PDOs with the indicated concentration of RSPO1 or RSPO3 relative to 1000 ng/ml RSPO1 was calculated. Each experiment was performed in triplicate and repeated more than three times.

### Three-dimensional co-culture assay

For direct 3D co-culture, GFP-labeled PDOs were trypsinized into single cells and seeded with or without CAFs at a 1:20 ratio (5 × 10^3^ PDO cells and 1 × 10^5^ CAFs/well) into a 24-well plate with Matrigel with serum medium. For indirect 3D co-cultures, CAFs were seeded into the trans-well membrane (3 μm pore size, 353,096; Corning) with Matrigel. Fluorescence and phase-contrast images of PDOs with or without CAFs were captured using BZ-X700 with the Z-stack and image stitching function at day 10. Fully focused and overlaid images were generated using BZ-X Analyzer. The total area of PDOs for each well was measured using the HybridCellCount software module of BZ-X Analyzer.

### Drug treatment assays

PDOs were dissociated into single cells and 5,000 cells were seeded into a 24-well plate with Matrigel. For gemcitabine treatment, PDOs cultured in combined medium or serum medium for 7 days were treated with the indicated concentration of gemcitabine (Gemzar; Eli Lilly Japan K.K., Kobe, Hyougo, Japan) for 72 h. For simvastatin treatment, immediately after seeding with Matrigel, cells were treated for 10 days in combined medium or serum medium with the indicated dose of simvastatin (S6196; Sigma Aldrich). PDOs were then trypsinized into single cells and cell viability for each well was quantified using the CellTiter-Glo Kit as described above.

### Immunohistochemistry and immunofluorescent staining

PDOs isolated from Matrigel using Cell Recovery Solution (354,253; Corning) were embedded in iPGell (PG20-1; GenoStaff, Tokyo, Japan) without damaging the 3D structures according to the manufacturer’s protocol. PDOs were then fixed with 4% paraformaldehyde and used to create paraffin-embedded blocks. Paraffin-embedded blocks of PDOs and PDAC tissues were sectioned (4 μm) and subjected to standard hematoxylin and eosin (H&E) staining and immunostaining. The following primary antibodies were used: E-cadherin (ab15148; abcam, Cambridge, UK, 1:30), Actin (ab130935; abcam, 1:100), CK19 (sc25724; Santa Cruz Biotechnology, Inc., Dallas, TX, USA, 1:50), αSMA (M0851; Dako, Santa Clara, CA, USA, 1:200), and RSPO3 (17,193–1-AP; proteintech, Rosemont, IL, USA, 1:100). The secondary antibodies were EnVision + System-HRP Labelled Polymer (K4003; Dako) and Alexa Fluor 488 anti-rabbit (A11034; ThermoFisher) and 546 anti-mouse (A11030; ThermoFisher). Nuclei were counterstained with hematoxylin or 4′,6-diamidino-2-phenylindole (DAPI; Dojindo, Kumamoto, Japan). For immunohistochemistry, staining was developed with 3,3′-diaminobenzidine substrate chromogen (11,209-1A; Kanto Kagaku, Tokyo, Japan) Bright field images were acquired using BZ-X700. The stain-positive area was quantified using HybridCellCount software module of BZ-X Analyzer (Keyence).

### Transfection of fluorophores and small hairpin RNA

To obtain RFP-labeled CAFs and GFP-labeled PDOs, RFP and GFP lentiviral particles (RFP, LVP023-PBS; GFP, LVP001-PBS; GenTarget Inc., San Diego, CA, USA) were transfected into immortalized CAFs (CAF-1) and PDOs, respectively. RFP transfection into CAF-1 was performed according to the manufacturer’s instructions. GFP transfection into PDOs was performed as previously described [31]. Briefly, single-cell suspensions of PDOs with combined medium and viral particles were plated into Matrigel-coated 24-well plates and incubated at 37 °C. The next day, the medium with viral particles was carefully removed and fresh Matrigel and medium were overlaid on the cells attached to Matrigel. When PDOs became sub-confluent, blasticidin S hydrochloride (15,205; Sigma-Aldrich) was used to select GFP and RFP clones. RFP-positive CAFs and GFP-positive PDOs were sorted by the Cell Sorter SH800S (Sony Corporation, Tokyo, Japan). To obtain RSPO3-knockdown CAFs, two high-titer lentiviral particles packing small-hairpin RNA (shRNA) against RSPO3 (MISSION Lentiviral Transduction Particles; shRSPO3-1, TRCN0000056663; shRSPO3-2, TRCN0000373388; Sigma-Aldrich) or non-targeting shRNA (SHC016V; Sigma-Aldrich) as control were transfected into immortalized CAFs (CAF-1) according to the manufacturer’s instructions. Puromycin (631,305; Takara) was used for more than 3 weeks to select RSPO3 shRNA–expressing cells. Knockdown efficacy of RSPO3 shRNA was confirmed by real-time quantitative reverse transcription-PCR (qRT-PCR).

### Collection of conditioned media from PDOs (PDO-CM)

PDOs cultured in combined medium for 7 days were washed twice with PBS and the fresh basic medium was replaced. After 48 h incubation at 37 °C, the medium was collected and filtered with a 0.22-μm syringe filter (Z359904; Merck, Darmstadt, Germany). After centrifugation at 1,500 rpm for 5 min, the supernatants were collected and FBS was added to a concentration of 5%. We prepared serum medium as a control medium.

### RNA extraction and quantitative RT-PCR

RNA was extracted from CAFs cultured under different conditions (2D-culture with serum medium, 3D-culture with serum medium, and 3D-culture with PDO-CM) for 72 h and RFP-positive CAFs directly or indirectly co-cultured with PDO585 in serum medium for 10 days using a High Pure RNA Isolation kit (11,828,665,001; Roche) and DNase I (Roche) treatment according to the manufacturer’s instructions. RFP positive CAFs co-cultured with PDO585 were sorted by the Cell Sorter SH800S. Real-time qRT-PCR was performed using the iTaq Universal SYBR Green One-Step Kit (172–5150; BioRad, Hercules, CA, USA) and CFX96 Touch Real-Time PCR Detection systems (Bio-Rad). Transcript quantities were determined using the ΔΔCt method and values were normalized to GAPDH mRNA. The following primers purchased from Takara Bio (Kusatsu, Japan) were used in this study: RSPO1, 5′-TCCAGAGCTCCCAGTGGACA-3′ (forward) and 5′-CAGGTCACCAGCAGTCCTCAAG-3′ (reverse); RSPO2, 5′-AGAAGCCCAAACTGCCTTTGA-3′ (forward) and 5′-TCTGTAGCTGGCCTGTGAAACTG-3′ (reverse); RSPO3, 5′-CATGACAATGGTGGCAAATGAC-3′ (forward) and 5′-TTTAGCATCAAGGATTCAGACCT-3′ (reverse); RSPO4, 5′-AGCAAGTCTGTCCTCACTGCCTATC-3′ (forward) and 5′-CGGCAAATACAAATCCCGTTTC-3′ (reverse); GAPDH, 5′-GCACCGTCAAGGCTGAGAAC-3′ (forward) and 5′-TGGTGAAGACGCCAGTGGA-3′ (reverse).

### Statistics

Statistical analyses were performed using Prism7 (GraphPad, San Diego, CA, USA). Data are represented as the mean ± standard error of the mean (SEM) unless otherwise indicated. For the comparisons of two groups, the unpaired two-tailed Student’s t-test was performed, and a *P* value < 0.05 was considered to be statistically significant. The Kaplan–Meier analysis was used to analyze survival, with curves compared using the Log-rank (Mantel–Cox) test. Area under curve (AUC) values were calculated from a log (drug) vs. response curve with robust fit.

## Results

### Tumor differentiation grade is an important prognostic factor in PDAC

To evaluate the correlation between the differentiation grade and prognosis in PDAC, 242 PDAC patients were divided into two groups according to histopathological differentiation grading. Kaplan–Meier analysis revealed that moderately and poorly differentiated PDAC was associated with shorter postoperative survival compared with well differentiated PDAC (*P* = 0.0046, Fig. S1, Table S1). These results indicate that the tumor differentiation grade of PDAC has a significant impact on prognosis.

### Establishment of human PDOs of each differentiation grade

**Fig. 1:**
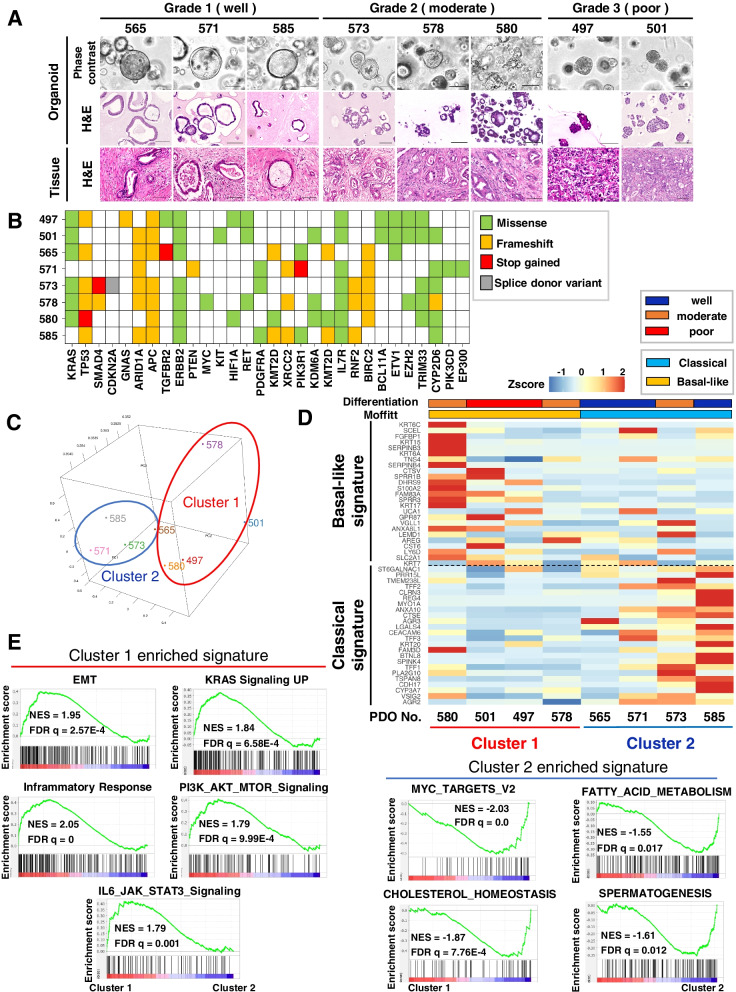
The expression profiles of PDOs differed depending on the differentiation grade. **A** Representative phase-contrast images (top) and H&E staining of PDOs from Grade1, Grade2, and Grade3 tumors (middle). H&E staining of the resected tumors from which the organoids were established (bottom). Scale bars, 100 µm. **B** Targeted genome sequencing analysis of PDOs. The type of mutation in the indicated genes is denoted by a color-coded key. **C** PCA plots for the transcriptome of PDOs isolated from different grades. PDOs were classified into two cluster groups, Cluster1 (red) and Cluster2 (blue). **D** Heatmap of gene expression levels according to Moffitt’s “Classical” and “Basal-like” signatures in PDOs. The two bars indicate tumor differentiation (top) and Moffitt’s classification (bottom). **E** HALLMARK pathways enriched by GSEA among differentially expressed genes between Cluster1 and Cluster2

To identify the molecular characteristics of each differentiation grade in PDAC, we established eight PDOs from resected specimens from PDAC patients. For histopathological analysis, we performed hematoxylin and eosin staining of the primary tumors and PDOs (Fig. 1A). PDO565, PDO571, and PDO585 formed well-defined ductal structures and were classified as well differentiated (Grade1) PDAC. PDO573, PDO578, and PDO580 comprised small irregular fused glands and were classified as moderately differentiated (Grade2) PDAC. PDO497 and PDO501, in which the primary tumor showed a cord-like arrangement, presented solid structures and were classified as poorly differentiated (Grade3) PDAC. All established PDOs reflected the morphological features of the primary tumor and could be classified into each differentiation grade by histopathological evaluation.

### Genomic and transcriptomic characterization of PDOs

To compare the tumor genomic background with differentiation grade, we performed targeted genome sequencing of PDOs (Fig. 1B). All PDOs except PDO571 harbored *KRAS* or *TP53* mutations, which are commonly reported in PDAC. PDO571 did not have *KRAS* or *TP53* mutations but harbored *ARID1A* and *EP300* mutations, as well as mutations in *PIK3CD*, *PIK3R1*, and *PTEN*, which are related to the PI3K pathway [32]. However, no gene mutations correlated with the differentiation grade.

To characterize transcriptional phenotypes of differentiation grades, we next performed gene expression microarray analysis of PDOs. Three-axis principle component analysis classified PDOs into two major clusters (Fig. 1C). Cluster1 included PDO578 and PDO580 with Grade2, and PDO497 and PDO501 with Grade3, whereas Cluster2 included PDO565, PDO571, and PDO585 with Grade1 and PDO573 with Grade2. Using Moffitt’s classification [17], Cluster1 was classified as “Basal-like” and Cluster2 as “Classical” (Fig. 1D). GSEA revealed that genes related to epithelial-mesenchymal transition (EMT), inflammatory pathway and cell proliferation, such as KRAS and PI3K/AKT/mTOR signaling pathways, were upregulated in Cluster1, which contains all Grade3 PDOs (Fig. 1E). In Cluster2, which mainly comprised Grade1 PDOs, genes related to MYC, a Wnt pathway target gene, as well as fatty acid metabolism and cholesterol homeostasis were upregulated. These results indicate that Grade1 PDOs were classified as “Classical” and Grade3 PDOs were classified as “Basal-like” and these groups exhibited distinct expression signatures.

### Microenvironment factors are essential for the differentiated PDO phenotype

**Fig. 2:**
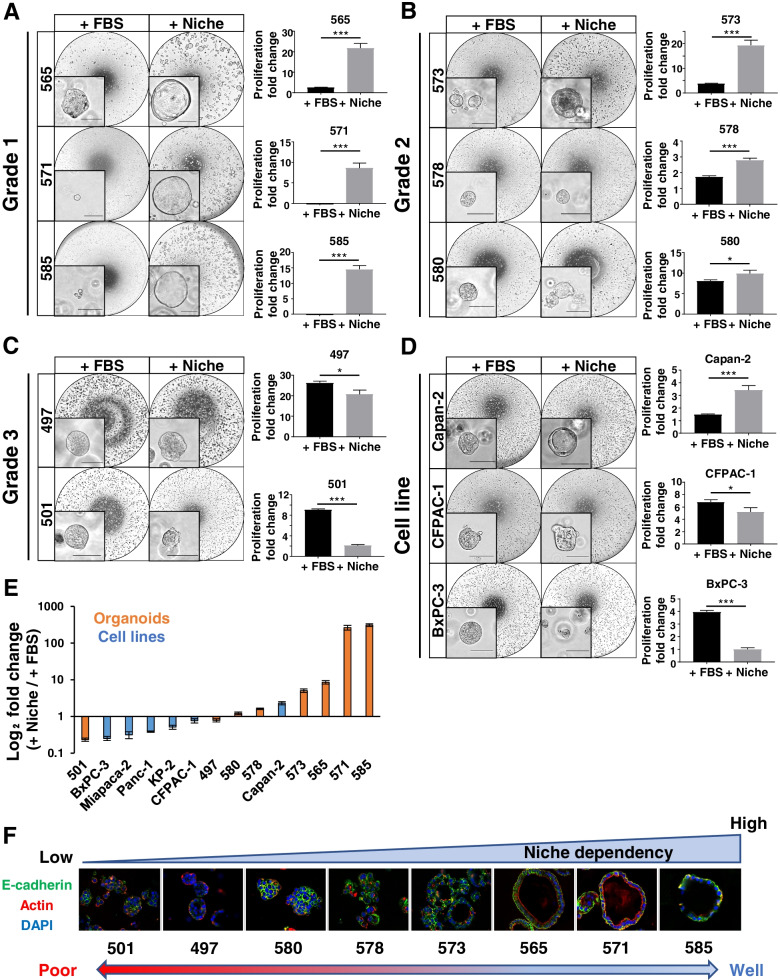
Microenvironmental factors are essential for the growth and ductal formation of well differentiated PDO. **A**–**D** Effect of niche factors on the proliferation and differentiation of PDOs and PCCs. (left) Representative images of Grade1 (**A**), Grade2 (**B**), Grade3 (**C**) PDOs, and PCCs (**D**) cultured in serum medium or niche medium. Serum medium comprised basic medium supplemented with only 5% fetal bovine serum (FBS). Niche medium comprised basic medium supplemented with niche factors. + FBS, serum medium; + niche, niche medium. Inset, highly magnified views show morphological features. Scale bars, 100 µm. (right) Quantification of the growth rate of PDOs and PCCs (*P < 0.05; **P < 0.01; ***P < 0.001). **E** Summary of niche dependency in PDOs and PCCs. Niche dependency scores were calculated as the ratio of the proliferation fold change in niche medium to that in serum medium. **F** Immunofluorescence images for actin (red), E-cadherin (green), and DAPI (blue) in PDOs cultured in combined medium, which comprised the basic medium supplemented with both niche factors and 5% FBS

Although no gene mutation significantly correlated with the differentiation grade, there were distinct differences in gene expression levels, which suggests that exogenous factors may contribute to molecular features. Therefore, we focused on microenvironmental factors (niche factors) added in PDO culture and evaluated proliferation and organoid formation in serum medium or niche medium (Fig. 2A–C). In Grade1 PDOs, almost no organoid formation and proliferation was observed in serum medium. Although several organoids proliferated in PDO565, they showed solid structures (Fig. 2A). In Grade2 PDOs, proliferation rates were higher in niche medium, but PDOs proliferated sufficiently in serum medium (Fig. 2B). The morphology of Grade2 PDOs in serum medium was similar to that of the Grade3 PDO, but in niche medium, the organoids formed slightly irregular ductal structures, a moderately differentiated form. In Grade3 PDOs, proliferation rates were significantly higher in serum medium (Fig. 2C). Grade3 PDOs showed solid structures even in niche medium. All PCCs except for Capan-2 showed higher proliferation in serum medium, as did Grade3 PDOs, and formed solid structures in both media (Fig. 2D). Only Capan-2 cells showed higher proliferative capacity in niche medium and showed ductal structures in niche medium. To clarify the effect of serum on PDOs, we additionally evaluated the effect of basic medium and niche medium with serum on proliferation (Fig. S2) In Grade1 PDOs, almost no organoid formation and proliferation was observed in basic medium as well as in serum medium. Moreover, the addition of serum to niche medium slightly inhibited the growth of Grade 1 PDOs, suggesting that serum has an inhibitory effect on niche dependent PDO growth. Therefore, the response of PDOs to serum differed according to the subtypes based on Moffitt's classification.

From these results, we evaluated the niche factor dependency as the ratio of proliferation values in niche medium to that in serum medium (Fig. 2E). Differentiated PDOs classified as Cluster2 and Capan-2 cells were highly dependent on niche factors, while Cluster1, including Grade3 PDOs and most PCCs, were less dependent on niche factors. To investigate the relationship between the niche factor dependency and cancer stem cell populations, we evaluated the correlation between niche factor dependency and the expression levels of PDAC stem cell markers including CD44, CD24, and CD133 [33, 34]. However, no significant correlation was found between the niche factor dependency and the expression levels of these stem cell markers (Fig. S3). Moreover, fluorescence immunostaining of PDOs cultured in combined medium showed that PDOs with higher niche factor dependency formed ductal structures while those with lower dependency showed solid structures (Fig. 2F). Therefore, niche factor dependency decreased as the degree of tumor differentiation changed from well to poorly differentiated, and Grade3 PDOs could be cultured in serum medium similar to PCCs. Additionally, niche factors were essential for forming ductal structures, a characteristic of differentiated PDAC.

### Well differentiated PDACs are closely surrounded by CAFs

**Fig. 3:**
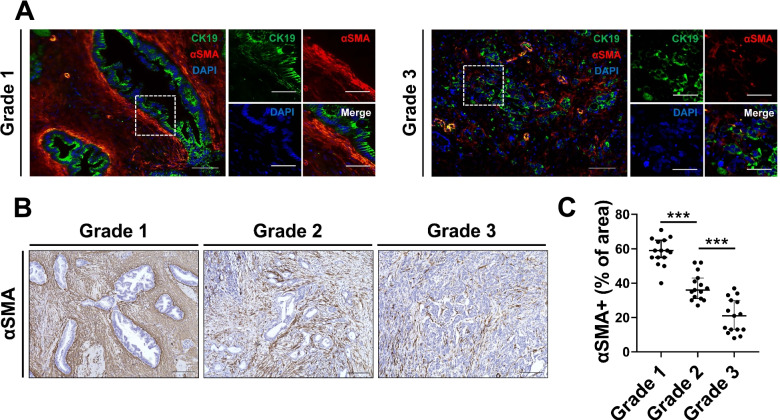
Grade1 PDAC is closely surrounded by abundant CAFs. **A** Representative immunofluorescence images for αSMA (red), CK19 (green), and DAPI (blue) in Grade1 and Grade3 portions of human PDAC. Scale bars, (left) 100 µm and (right) 50 µm. **B** Representative immunohistochemistry images for αSMA in surgically resected PDAC tissue of indicated tumor grades. Scale bars, 200 µm. **C** Quantification of αSMA staining area as a percentage of the total area in PDAC tissue of indicated tumor grades (*n* = 15). Data are presented as the mean ± SD (***P < 0.001)

Although niche factors were essential for differentiated PDOs, the cells supplying these niche factors in primary PDAC tissues were unknown. We focused on CAFs, which account for a large proportion of PDAC stroma, and investigated the relationship between the differentiation grade and the distribution of CAFs by immunofluorescence staining (Fig. 3A). In Grade1 PDAC, CAFs were widely distributed in the tumor stroma; CAFs strongly expressing αSMA lined the PDAC cells. In Grade3, the distribution of αSMA-positive CAFs was relatively small, and there was no obvious pattern in the spatial relationship between PDAC cells and CAFs. Quantification of the area of αSMA-positive CAFs by immunohistochemistry revealed that the amount of αSMA-positive area decreased significantly as the tumor grade increased from Grade1 to Grade3 (Fig. 3B and C, E).

### Well differentiated PDOs are strongly dependent on CAFs for growth, and CAFs maintain the ductal structure of PDOs

**Fig. 4:**
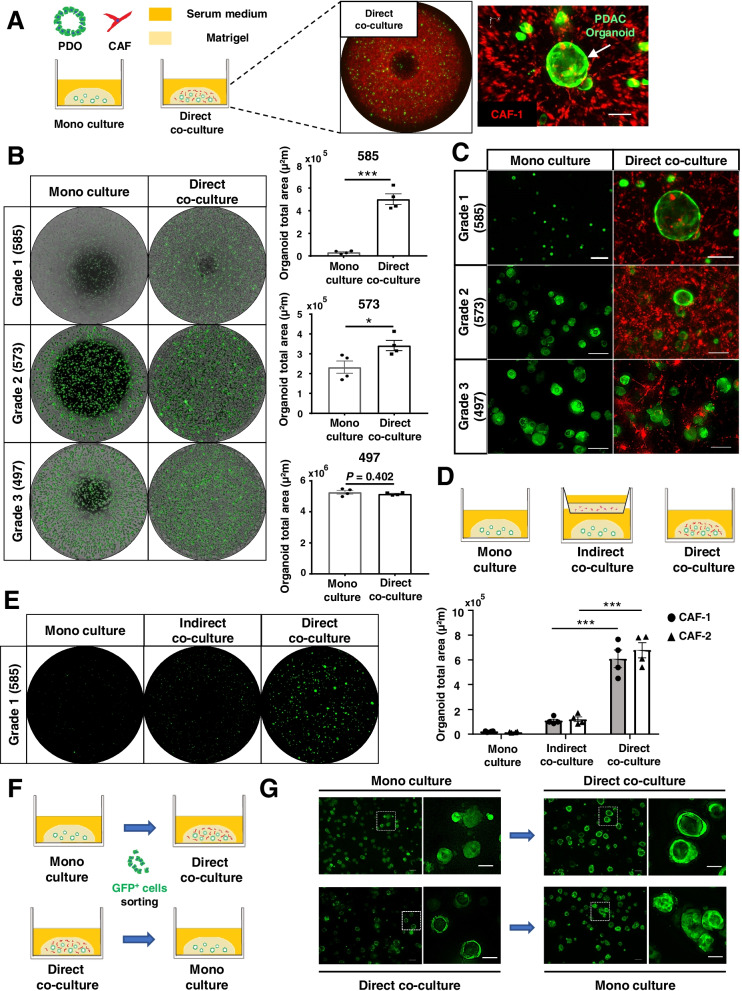
CAFs have substantial effects on the growth and morphology of well and moderately differentiated PDOs. **A** Schema of 3D mono- and direct co-culture models, and representative fluorescence images of 3D direct co-culture model: low magnified view (left), high magnified 3D reconstruction view (right). PDO (green), CAF-1 (red). Scale bar, 100 µm. **B** Representative fluorescence images overlaid with phase contrast images of PDOs (green) cultured with or without CAF-1, and quantification of the total area of PDOs (*P < 0.05; **P < 0.01; ***P < 0.001). **C** Representative high magnified fluorescent images of (**B**) showing morphological features. Scale bars, 100 µm. **B** Schema of the 3D mono-, indirect and direct co-culture models. **E** Representative fluorescence images of Grade1 PDOs mono-cultured and co-cultured indirectly or directly with CAF-2, and quantification of the total area of PDOs with CAF-1 and CAF-2 (***P < 0.001). **F** Schematic diagram of the protocol to evaluate morphological features of Grade2 PDOs in the presence or absence of CAFs. **G** Representative fluorescence images of Grade2 PDOs (573) mono-cultured or co-cultured with CAF-1, before sorting (left) and after sorting (right). Scale bars, 100 µm (low magnification), 50 µm (high magnification)

The relationship between the differentiation grade and CAFs surrounding PDAC cells suggests that CAFs supply the niche factors essential for differentiated PDAC. We generated a 3D co-culture model of PDOs and CAFs (Fig. 4A) and evaluated how CAFs affect the organoid-formation ability and morphology (Fig. 4B and C). Similar to the previous results, Grade1 PDOs did not show organoid formation in serum medium. However, when directly co-cultured with CAFs, Grade1 and Grade2 PDOs significantly formed organoids with ductal structures, as under the supplementation of niche factors. Moreover, Grade2 PDOs with CAFs showed ductal structures, whereas Grade2 PDOs without CAFs showed solid structures. In contrast, Grade3 PDOs showed no differences in organoid formation with or without CAFs, and their morphology remained unchanged as solid structures. Thus, direct co-culture of PDOs with CAFs led to the same results as under the supplementation of niche factors, which suggests that CAFs are a source of niche factors Additionally, the indirect co-culture of Grade1 PDOs with CAFs showed almost no organoid formation, as in monoculture (Fig. 4D and E). However, supplementation of niche factors to Grade1 PDOs following indirect co-culture with CAFs for 7 days allowed organoid formations, suggesting that the expression levels of CAF-derived niche factors are so minimal to affect only the proximity of CAFs (Fig. S4).

We next examined whether the morphological differences between Grade2 PDOs with and without CAFs were from the heterogeneity of organoids with different differentiation grades or the plasticity to change their morphology depending on CAFs. Therefore, after monoculture or co-culture of PDOs with CAFs, the PDOs were sorted by flow cytometry and cultured again under other conditions to observe the morphological changes (Fig. 4F). The Grade2 PDOs showed plasticity to change morphology depending on CAFs, as they showed ductal structures only in direct co-culture with CAFs and solid structures in monoculture (Fig. 4G). These results indicate that CAFs play an integral role in maintaining the differentiated phenotype in PDAC.

### Differentiated PDOs are strongly dependent on RSPO

**Fig. 5:**
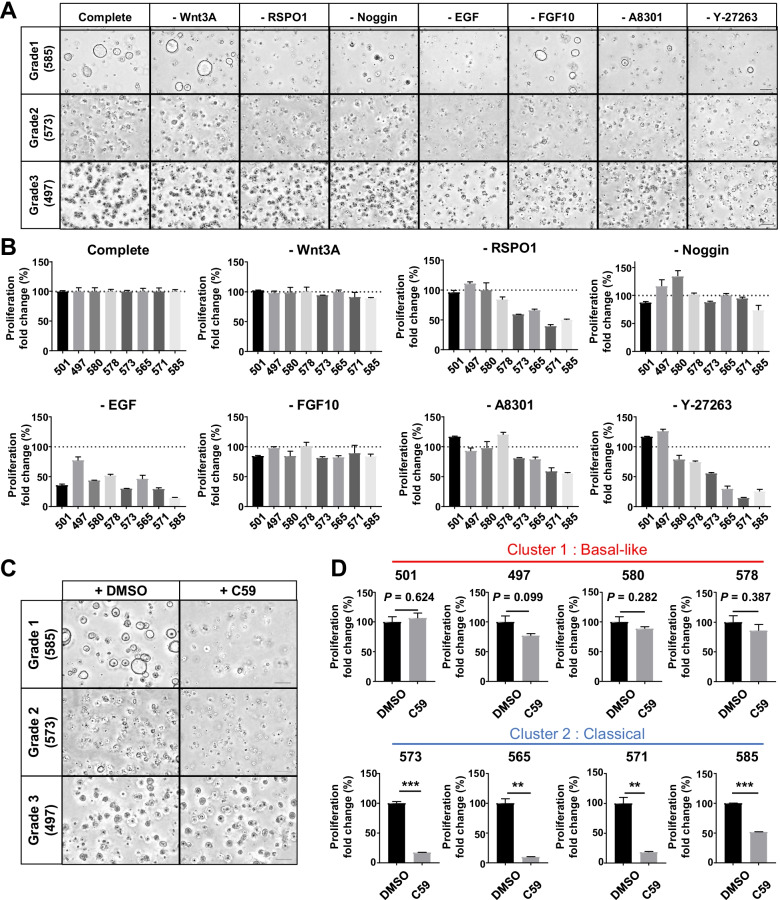
Differentiated PDOs are dependent on autocrine Wnt and exogenous RSPO. **A** Representative phase contrast images of Grade1, Grade2, and Grade3 PDOs cultured in niche medium or medium lacking the indicated factors. Scale bars, 100 µm. **B** Proliferation rate of PDOs cultured in the absence of the indicated factors compared with growth in full niche medium. **C** Representative phase contrast images of Grade1, Grade2, and Grade3 PDOs treated with vehicle (DMSO) or Porcn-inhibitor (C59) in niche medium lacking Wnt3A. Scale bars, 200 µm. **D** Proliferation rate of PDOs treated with C59 compared with PDOs treated with DMSO (**P < 0.01; ***P < 0.001)

The above results suggest that CAFs maintain the differentiated PDO phenotype by secreting niche factors. However, PDOs can not be formed in indirect co-culture with CAFs, suggesting that the expression levels of factors supporting organoid formation secreted by CAFs can be minimal that function only in the specific local space near interacted cells. Therefore, the present study focused on the minimal and niche factors produced by CAFs and narrowed down the candidates from the known niche factors for organoid culture (organoid niche factors) that showed a similar response in direct co-culture with CAFs. To determine which factor among organoid niche factors was most critical for PDO phenotype, we next evaluated the influence of each niche factor on the growth of PDOs (Fig. 5A and B). PDAC is classified into three subtypes based on dependency on Wnt signals: “Wnt non-secreting,” “Wnt secreting,” and “Wnt independent [35].” All PDOs in this study were not dependent on exogenous Wnt. We also examined the effect of a Porcn-i (C59) that inhibits the production of active Wnt ligands to determine whether the PDOs autonomously produce their own Wnt niche [36]. Notably, C59 significantly inhibited the proliferation of only differentiated PDOs classified as Cluster2, “Classical” subtype (Fig. 5C and D). Additionally, the removal of RSPO1, a family of secreted molecules that strongly potentiate Wnt/β-catenin signaling through stabilization of Wnt receptors [37], also compromised the growth of differentiated PDOs classified as Cluster2, “Classical” subtype (Fig. S5). These results suggest that differentiated PDOs classified as Cluster2 were dependent on the Wnt pathway via autocrine Wnt and exogenous RSPO for their proliferation. Moreover, omission of ROCK inhibitor did not affect the proliferation of Grade3 PDOs classified as Cluster1, “Basal-like” subtype, which suggests that these PDOs had already acquired resistance to anoikis (Fig. S5). We selected RSPO as a candidate of CAF-derived factors that contribute to differentiated PDO formation among organoid niche factors based on these results,

### CAFs provide RSPO3 to well differentiated PDAC and contribute to growth

**Fig. 6:**
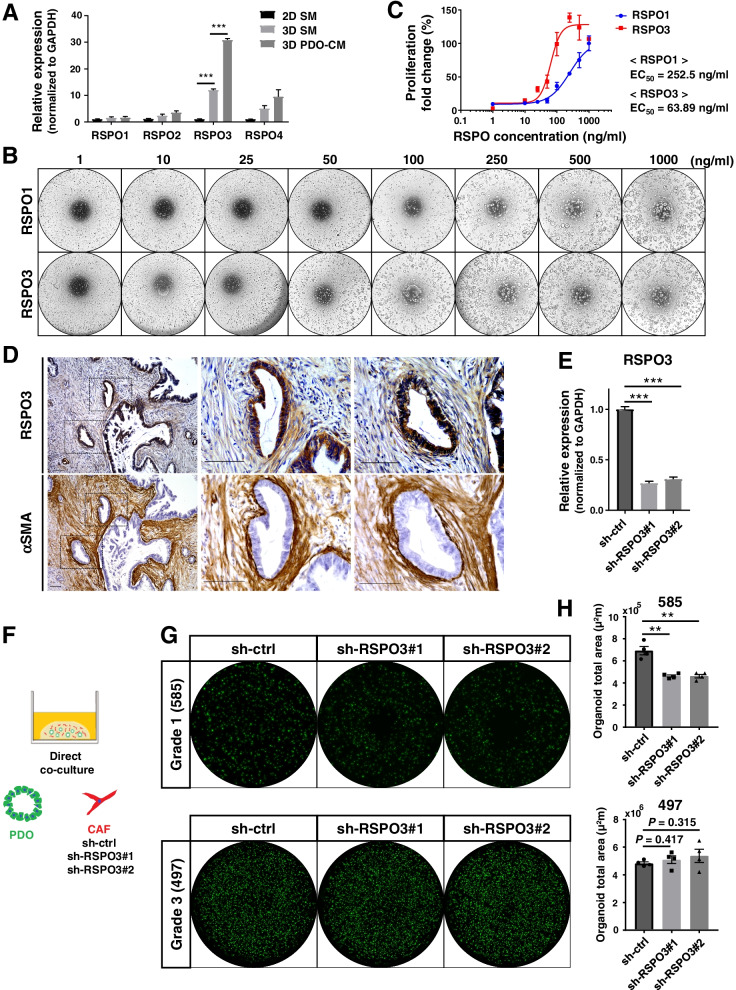
CAF-derived RSPO3 contributes to the growth of well differentiated PDAC with high dependency on RSPO. **A** qRT-PCR analysis of the mRNA expressions of RSPO1–4 in CAFs under different conditions (2D-culture with serum medium, 2D SM; 3D-culture with serum medium, 3D SM; 3D-culture with PDO conditioned serum medium, 3D PDO-CM). Results are shown relative to the expression in CAFs 2D-cultured with serum medium after normalization by GAPDH mRNA expression (***P < 0.001). **B** Representative phase contrast images of Grade1 PDOs (585) cultured in niche medium with the indicated concentration of recombinant RSPO1 or RSPO3. **C** Dose–response curve of Grade1 PDOs to RSPO1 and RSPO3. Results were normalized using the proliferation of PDOs with RSPO1 at 1000 ng/ml as 100%. **D** Representative images of immunohistochemical staining for RSPO3 and αSMA in serial sections of human Grade1 PDAC tissue. Scale bars, 100 µm. **E** Transfection of RSPO3-targeting shRNA (sh-RSPO3#1 and sh-RSPO3#2) decreased RSPO3 mRNA expression in CAFs compared with negative control shRNA (sh-ctrl) as confirmed by qRT-PCR analysis (***P < 0.001). **F** Schema of 3D direct co-culture models using PDOs and shRNA-transfected CAFs. **G** Representative fluorescence images of Grade1 and Grade3 PDOs directly co-cultured with shRNA-transfected CAFs. **H** Quantification of the total area of PDOs direct co-cultured with shRNA-transfected CAFs (**P < 0.01)

RSPOs (also known as roof plate-specific-spondins) are a family of four secreted proteins (RSPO1–4) in vertebrates, and all four RSPO proteins stimulate Wnt signaling [38]. To identify the candidate among CAF-derived RSPOs that supports differentiated PDAC growth, we first evaluated the effect of PDAC cells on the expression of RSPO members in CAFs. Exposure of PDO supernatant significantly increased the mRNA expression of only RSPO3 (Fig. 6A). We then examined the effects of RSPO3 on PDO proliferation using RSPO3 recombinant protein compared with RSPO1 (Fig. 6B). Recombinant RSPO3 promoted the proliferation of Grade1 PDOs at a significantly lower dose than RSPO1, with EC_50_ of 63.89 ng/ml and 252.5 ng/ml, respectively (Fig. 6C). These results indicate that RSPO3 is the CAF-derived RSPO that functions to promote differentiated PDAC growth.

To identify the localization of RSPO3 protein in primary PDAC tissue, we next performed immunohistochemistry for RSPO3 and αSMA using serial sections (Fig. 6D). PDAC cells and spindle-shaped cells lining the cancer cells were stained positive for RSPO3 in Grade1 PDAC tissue. In serial sections, the spindle-shaped cells were also stained positive for αSMA, which suggests that CAFs proximal to the cancer cells secreted RSPO3.Furthermore, we compared the expression level of RSPO3 in CAFs between indirect and direct co-culture because Grade1 PDOs showed almost no organoid growth in indirect co-culture with CAFs. Then, we found that there was no significant difference in RSPO3 expression between indirect and direct co-culture, suggesting that contact between PDOs and CAFs does not affect the level of RSPO3 secretion (Fig. S6).

To investigate whether CAF-derived RSPO3 maintains the growth of differentiated PDAC, we generated CAFs transfected with two shRNAs targeting RSPO3 or control shRNA (Fig. 6E). Grade1 and Grade3 PDOs were then directly co-cultured with these CAFs in serum medium to compare organoid formation (Fig. 6F). In Grade1 PDOs, which are highly dependent on RSPO, organoid formation was significantly inhibited when PDOs were co-cultured with RSPO3-knockdown CAFs compared with control CAFs (*P* < 0.005, Fig. 6G). In contrast, organoid formation of Grade3 PDOs, which are independent of RSPO, was not affected when co-cultured with RSPO3-knockdown CAFs (Fig. 6H). These results suggest that CAF-derived RSPO3 supports the growth of Grade1 PDAC.

### PDO subtypes based on niche factor dependency show distinct drug treatment responses

**Fig. 7:**
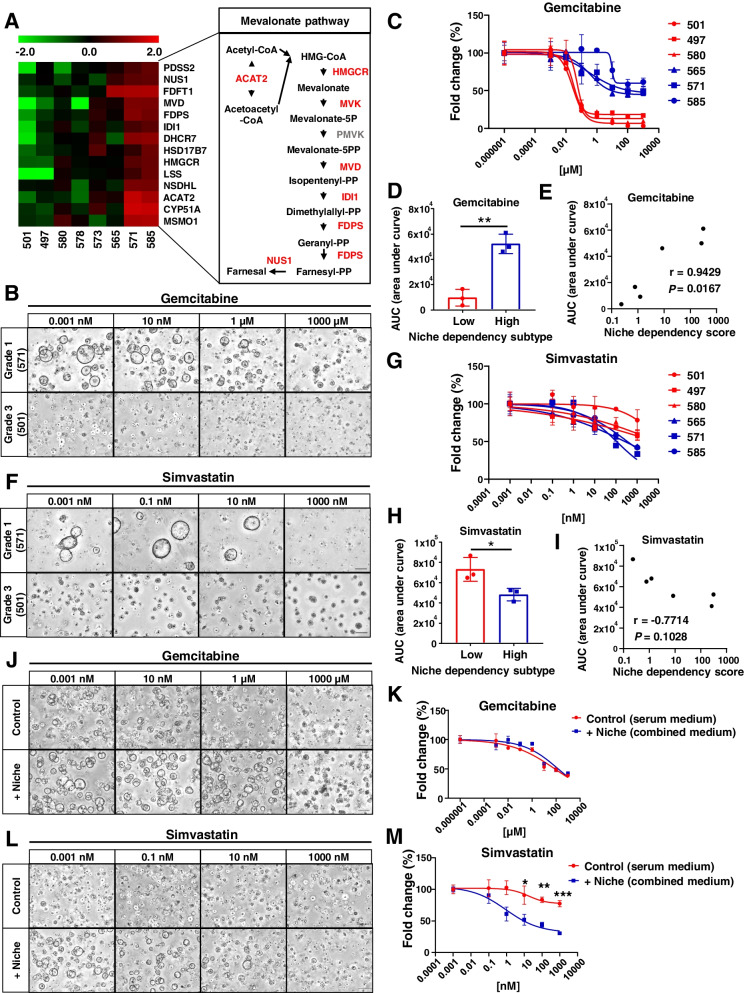
The PDO subtypes based on niche factor dependency show distinct drug treatment responses. **A** Heatmap of expression levels on the genes of mevalonate pathway–related enzymes in PDOs (left). Simplified overview of the mevalonate pathway (right). Upregulated enzymes in PDOs with high niche dependency are shown in red. **B** Representative images of PDOs treated with the indicated concentration of gemcitabine for 72 h after 7 days’ culture in combined medium. Scale bars, 200 µm. **C** Dose–response curve of PDOs treated with gemcitabine for 72 h. Results were normalized using the proliferation of PDOs treated with gemcitabine 0.001 nM as 100% response. **D** AUC distribution of gemcitabine sensitivity in the “High” and “Low” niche dependency subtypes (**P < 0.01). **E** Spearman’s correlation analysis between niche dependency and sensitivity to gemcitabine. **F** Representative images of PDOs cultured in combined medium with the indicated concentration of simvastatin for 10 days. Scale bars, 200 µm. **G** Dose–response curve of PDOs treated with simvastatin for 10 days. Results are normalized according to the proliferation of PDOs treated with simvastatin 0.001 nM as 100% response. **H** AUC distribution of simvastatin sensitivity in the “High” and “Low” niche dependency subtypes (*P < 0.05). **I** Spearman’s correlation analysis between niche dependency and sensitivity to simvastatin. **J** Representative images of PDO573 treated with the indicated concentration of gemcitabine for 72 h after 7 days’ culture in serum medium or combined medium. Scale bars, 200 µm. **K** Dose–response curve of PDO573 treated with gemcitabine for 72 h. Results were normalized using the proliferation of PDO573 treated with gemcitabine 0.001 nM as 100% response. **L** Representative images of PDO573 cultured in serum medium or combined medium with the indicated concentration of simvastatin for 10 days. Scale bars, 200 µm. **M** Dose–response curve of PDOs treated with simvastatin for 10 days. Results are normalized according to the proliferation of PDOs treated with simvastatin 0.001 nM as 100% response

The present results suggest that the dependency on CAF-derived niche factors, such as RSPO3, varies with the differentiation grade in PDAC and that the molecular characteristics induced by CAF-derived niche factors may be new therapeutic targets. To identify the transcriptomic signatures induced by niche factors, we extracted genes positively or negatively correlated with the niche dependency scores from the transcriptome data (Fig. S7A and B). Kyoto Encyclopedia of Genes and Genomes (KEGG) pathway analysis showed that expression of genes related to the terpenoid backbone and steroid biosynthetic pathways were upregulated as the niche dependency increased, while the expression of genes related to the proteasome and cell cycle was upregulated as the niche dependency decreased (Fig. S7C). Furthermore, among the positively correlated genes, the gene expression of enzymes involved in the mevalonate pathway was notably upregulated in PDOs with high niche dependency (Fig. 7A).

As shown above, PDOs have different molecular features according to niche dependency. Thus, we classified PDOs into two subtypes based on niche dependency (“High,” PDO565, PDO571, and PDO585; “Low,” PDO497, PDO501, and PDO580) and examined the differences in drug response. At first, targeting the cell cycle upregulated in the low niche dependency subtype, the therapeutic response to gemcitabine was evaluated (Fig. 7B). The low niche dependency subtype, in which cell cycle genes were upregulated, was significantly more sensitive to gemcitabine (*P* = 0.0019, Fig. 7C and D), while several PDOs in the high niche dependency subtype survived under high doses of gemcitabine. There was also a strong correlation between niche dependency and the sensitivity to gemcitabine (Fig. 7E). We next investigated the response to simvastatin, an hydroxymethylglutaryl-CoA (HMG-CoA) reductase inhibitor, in each subtype (Fig. 7F). In the high niche dependency subtype, in which the mevalonate pathway is upregulated, the proliferation of PDOs was significantly inhibited by simvastatin (*P* = 0.0315, Fig. 7G and H). There was a correlation between niche dependency and response to simvastatin treatment (Fig. 7I).

In Fig. 4G, Grade 2 PDO573 showed differentiation plasticity, changing its morphology depending on the presence or absence of niche factors or CAFs. Therefore, we investigated whether this differentiation plasticity affects the treatment response. PDO573 was cultured in serum medium and combined medium to induce dedifferentiated and differentiated states, respectively, and then treated with gemcitabine and simvastatin. In the therapeutic response to gemcitabine, there was no significant difference in either medium (Fig. 7J and K). However, simvastatin significantly inhibited the proliferation of the differentiated state, which was induced by niche factors compared with the dedifferentiated state cultured under serum medium (Fig. 7L and M). Furthermore, under high concentration of statin, Grade 2 PDO showed a solid, dedifferentiated morphology even with niche factors.These results suggest that niche factors regulate the molecular phenotype of PDAC and indicate that the subtypes based on niche factor dependency showed distinct therapeutic responses.

## Discussion

Previous studies showed that stroma-targeting therapy in PDAC mouse models increased the proportion of poorly differentiated type cells [14, 15]. However, how the stroma regulates the PDAC phenotype has not been clarified. In this study, analyses using human PDOs revealed that CAFs supply niche factors and maintain the differentiated PDAC phenotype. The present data also suggest that these niche factors induce distinct molecular features that may represent novel therapeutic targets in differentiated PDAC.

The differentiation grade in PDAC is one of the critical clinical indicators [16] and, as shown in this study, the well differentiated type shows a significantly better prognosis than the poorly differentiated type. We classified PDOs into two clusters based on expression analysis. These clusters of PDOs corresponded to Moffitt’s classification of “Classical” and “Basal-like” using bulk PDAC tissues and had the same characteristics in pathway analysis as previously reported [17]. In this study, all well differentiated types and poorly differentiated types were classified as “Classical” and “Basal-like” types, respectively. Therefore, the morphological and molecular signatures of the PDO model are consistent with the bulk expression profiles of primary PDAC, which indicates that PDO is a useful in vitro model that reflects the characteristics of the primary tissue.

We considered that exogenous factors might determine the PDAC phenotype and thus focused on niche factors. Previous studies reported that the dependency of PDOs on niche factors was variable [22, 39] and PDOs acquired niche independency and higher signature scores of “Basal-like” type rather than “Classical” type through driver gene mutations and GATA6-mediated transcriptional reprogramming [35]. Consistent with the results, in this study, differentiated PDOs, which were classified as “Classical,” heavily depended on niche factors not only for proliferation but also for the formation of ductal structures, while Grade3 PDOs and PCCs except Capan-2, which were classified as “Basal-like [5],” acquired niche independency and formed solid structures even with niche factors. However, among the PDOs classified as “Classical,” PDO565, Grade2 PDOs, and Capan-2, a PCC established from a differentiated PDAC, showed plasticity that altered the differentiation grade depending on the presence or absence of niche factors. These results indicate that PDAC phenotypes are regulated not only by intracellular alterations of PDAC cells but also by exogenous factors.

PCCs are usually cultured in serum medium, which is critically different from the culture conditions of PDOs, there has been no reports that examined the response of PDOs to serum for each tumor grade. In the present study, the niche factor-dependent proliferation of Grade1 PDOs was decreased by serum addition, but that Grade2/3 PDOs proliferation was promoted by serum addition. Moreover, Grade3 PDOs, which were classified as “Basal-like,” similar to PCCs, proliferated better in serum medium. Thus, even PDOs could be cultured in serum medium if they were derived from high Grade PDAC tumor. Although it was reported that the addition of serum is detrimental to the PDO culture[5, 35], we found that serum addition is not detrimental to all PDOs but that the serum response varies depending on the tumor grade and Moffitt’s classification regardless of PCC or PDO.

Our study showed that Grade1 PDOs did not form organoids or proliferate in serum medium, but the addition of niche factors to serum medium caused organoid formation and proliferation, suggesting that serum does not contain enough niche factors required by Grade1 PDO. On the other hand, it was reported that there was an interaction between the tumor grade and the stroma in PDAC tissues, where low grade PDAC cells maintain activated stroma by reduced expression of CSF-1, suggesting that these PDAC cells benefit from the activated stroma-derived signals [40]. In the present study, differentiated PDOs with high niche dependency maintained the differentiated phenotype even in serum medium when co-cultured with CAFs, which suggests that differentiated PDAC is dependent on CAF-derived niche factors to maintain the differentiated phenotype. Additionally, Grade2 PDOs had the plasticity to change morphology into the differentiated or poorly differentiated type depending on the presence or absence of CAFs. These results indicate that suppression of CAFs could switch the PDAC phenotype from the differentiated to poorly differentiated type, which is consistent with the previous report of stroma-targeting therapy in PDAC mouse models [14, 15]. Furthermore, Grade3 PDOs and PCCs did not have the plasticity to switch to the differentiated types even in the presence of CAFs. These data suggest that conventional experimental systems using PCCs, which are mostly “Basal-like” and poorly differentiated types, are unsuitable for evaluating the switching of PDAC phenotypes.

Recent studies described three distinct populations of CAFs in PDAC: myofibroblastic CAFs (myCAFs), which have high αSMA expression, inflammatory CAFs (iCAFs), which express less αSMA but secrete more IL-6 and other inflammatory factors, and antigen-presenting CAFs (apCAFs), which express MHC class II and CD74 [41–43]. Although both CAFs used in this study expressed αSMA, it was difficult to distinguish precisely which CAFs they belonged to because no specific marker has been identified for their isolation. However, myCAFs are located in direct proximity to cancer cells, and recent studies reported that stromal myofibroblasts secrete RSPO3 to support gastrointestinal epithelial stem cells [44, 45]. In this study, immunohistochemistry showed that CAFs proximal to the cancer cells expressed RSPO3 along with αSMA, suggesting that a specific population of myCAFs located close to cancer cells maintain their differentiation signatures through secreting niche factors. On the other hand, the present expression analysis showed that IL-6-JAK-STAT pathway and inflammatory response were upregulated in high-grade PDOs, suggesting that iCAFs with high IL-6 expression may be involved in high-grade PDAC progression.

Our results indicated that the molecular features of PDOs varied according to niche factor dependency. Previous reports demonstrated that CAFs promoted prostate cancer progression through upregulation of cholesterol and steroid biosynthesis [46]. The present data also indicated that CAF-derived niche factors induced the expression of genes related to the mevalonate pathway and steroid biosynthesis in Grade1 PDAC, although this study did not clarify the direct expression changes affected by CAFs. Our findings also indicate that Grade1 PDOs are dependent on the Wnt pathway via autocrine Wnt and exogenous R-spondin, and Deng et al. showed that the mevalonate pathway was upregulated by Wnt/β-catenin signaling in pancreatic cancer [47]. These results suggest that differentiated PDAC depends on CAFs to upregulate the mevalonate pathway following activation of the Wnt pathway. Furthermore, in Grade 2 PDO with differentiation plasticity, statins significantly inhibited the proliferation of the differentiated state, which was induced by niche factors such as Wnt and RSPO compared with the dedifferentiated state. In addition, under high statin concentration, Grade2 PDO showed a solid, dedifferentiated morphology even with niche factors. Consistent with the results, it has been reported that inhibition of cholesterol pathway by statin in mouse PDAC cells increased high-grade PDAC [51]. Taken together, these data suggest that the mevalonate pathway is involved in the niche factor-mediated proliferation and differentiation of PDOs.

Statins are key drugs targeting the rate-limiting enzyme in the mevalonic acid/cholesterol synthesis pathway [48]. In vitro and in vivo pancreatic cancer models have shown that statins inhibit tumor growth by arresting the cell cycle and inhibiting DNA synthesis in G1 in cancer cells [49–51]. However, a phase II clinical trial revealed that gemcitabine and simvastatin combination therapy had no clinical benefit compared with gemcitabine alone [52]. Although clarifying the reasons underlying inconsistent results between preclinical and clinical studies can be challenging, data from the present study may provide one reason. If the statin combination therapy is applied to only the high niche dependency subtype, a clinical therapeutic effect of statin would be observed. Taken together, it is suggested that PDAC phenotype is regulated by the tumor microenvironment, such as CAF-derived niche factors, and that the difference in niche factor dependency causes distinct drug responses.

## Conclusions

Tumor differentiation in PDAC has a significant impact on prognosis. The results of our study suggest that differentiation grade of PDAC is maintained by niche factors such as RSPO3 derived from CAFs and subtypes based on the dependency on these niche factors show distinct drug treatment responses. While tumor grade and molecular subtypes are classifications of cancer cells only, subtypes based on niche factor dependency that are also related to the microenvironment have the potential to lead to the development of stromal-targeted therapy and subtype-based therapeutic strategies for PDAC.

## Supplementary Information

**Additional file 1: Fig. S1. **A lower degree of tumor differentiation correlates with poor prognosis in patients with pancreatic ductal adenocarcinoma (PDAC). Kaplan–Meier overall survival analysis based on histopathological tumor differentiation in patients with PDAC (*n* = 242). **Fig. S2.**  The serum response of PDOs differed according to the tumor grade and Moffitt‘s classification. **Fig. S3.** Spearman’s correlation analysis between the niche factor dependency and the expression levels of PDAC stem cell markers, including CD44, CD24, and CD133. **Fig. S4.** Supplementation of niche factors to Grade1 PDOs following indirect co-culture with CAFs allowed organoid formations. **Fig. S5.** The relationship between Moffitt’s classification (“Classical”and“Basal-like”) and the proliferation rate of PDOs in the absence of the indicated factors (**P<0.01; ***P<0.001).  **Fig. S6.** qRT-PCR analysis of the mRNA expressions of RSPO3 in CAFs co-cultured indirectly or directly with PDO585. indirect, indirect co-culture; direct, direct co-culture. **Fig. S7.** Transcriptomic signatures induced by niche factors.

**Additional file 2: Table S1. **Relationship betweentumor differentiation and clinicopathologic factors.

## Data Availability

All data generated or analyzed during this study are included either in this article or in the supplementary information files. The genome sequencing data are deposited at Sequence Read Archive under accession numbers PRJNA755811 (http://www.ncbi.nlm.nih.gov/bioproject/755811). The microarray data are deposited at Gene Expression Omnibus under accession numbers GSE181593 (https://www.ncbi.nlm.nih.gov/geo/query/acc.cgi?acc=GSE181593).

## Abbreviations

AUC: Area under curve
CAFs: Cancer-associated fibroblasts
CI: Confidence interval
DAPI: 4′,6-Diamidino-2-phenylindole
DMEM: Dulbecco’s modified Eagle’s medium
DMSO: Dimethyl sulfoxide
EC_50_: Median effective concentration
EMT: Epithelial-mesenchymal transition
FBS: Fetal bovine serum
FDR: False Discovery Rate
GFAP: Glial fibrillary acidic protein
GSEA: Gene set enrichment analysis
H&E: Hematoxylin and eosin
HMG-CoA: Hydroxymethylglutaryl-CoA
HR: Hazard ratio
IL-6: Interleukin-6
KEGG: Kyoto Encyclopedia of Genes and Genomes
Mod: Moderately differentiated PDAC
MHC: Major histocompatibility complex
NES: Normalized enrichment score
PBS: Phosphate buffered saline
PCA: Principal component analysis
PCC: Pancreatic cancer cell line
PDAC: Pancreatic ductal adenocarcinoma
PDO: Patient-derived PDAC organoid
PDO-CM: Conditioned medium from PDOs
poor: Poorly differentiated PDAC
Porcn-i: Porcupine inhibitor
qRT-PCR: Quantitative reverse transcription polymerase chain reaction
RSPO: R-spondin
ShRNA: Small hairpin RNA
SM: Serum medium
TGF-β1: Transforming growth factor-β1
well: Well differentiated PDAC
αSMA: α-Smooth muscle actin
3D: Three-dimensional
