# Supplementary material for: Subtypes in pancreatic ductal adenocarcinoma based on niche factor dependency show distinct drug treatment responses
Source: J Exp Clin Cancer Res. 2022 Mar 10;41:89. doi: 10.1186/s13046-022-02301-9 (PMC8908673; doi:10.1186/s13046-022-02301-9)
Supplement: Supplementary file 1 — Additional file 1: Fig. S1. A lower degree of tumor differentiation correlates with poor prognosis in patients with pancreatic ductal adenocarcinoma (PDAC). Kaplan–Meier overall survival analysis based on histopathological tumor differentiation in patients with PDAC (n = 242). Fig. S2. The serum response of PDOs differed according to the tumor grade and Moffitt‘s classification. Fig. S3. Spearman’s correlation analysis between the niche factor dependency and the expression levels of PDAC stem cell markers, including CD44, CD24, and CD133. Fig. S4. Supplementation of niche factors to Grade1 PDOs following indirect co-culture with CAFs allowed organoid formations. Fig. S5. The relationship between Moffitt’s classification (“Classical”and“Basal-like”) and the proliferation rate of PDOs in the absence of the indicated factors (**P<0.01; ***P<0.001). Fig. S6. qRT-PCR analysis of the mRNA expressions of RSPO3 in CAFs co-cultured indirectly or directly with PDO585. indirect, indirect co-culture; direct, direct co-culture. Fig. S7. Transcriptomic signatures induced by niche factors. [file 13046_2022_2301_MOESM1_ESM.pdf]

Fig. S1

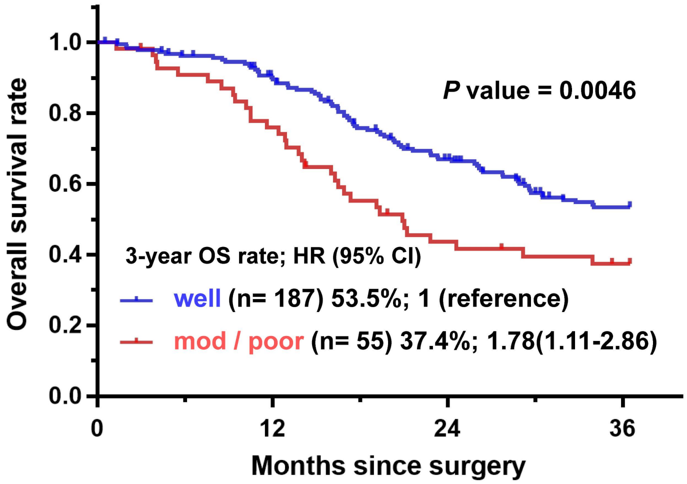

Fig. S1 A lower degree of tumor differentiation correlates with poor prognosis in patients with pancreatic ductal adenocarcinoma (PDAC). Kaplan–Meier overall survival analysis based on histopathological tumor differentiation in patients with PDAC (n = 242). P value and hazard ratio (HR) with 95% confidence interval (CI) were calculated using the log-rank test. well, well differentiated PDAC; mod, moderately differentiated PDAC; poor, poorly differentiated PDAC.

Fig. S2

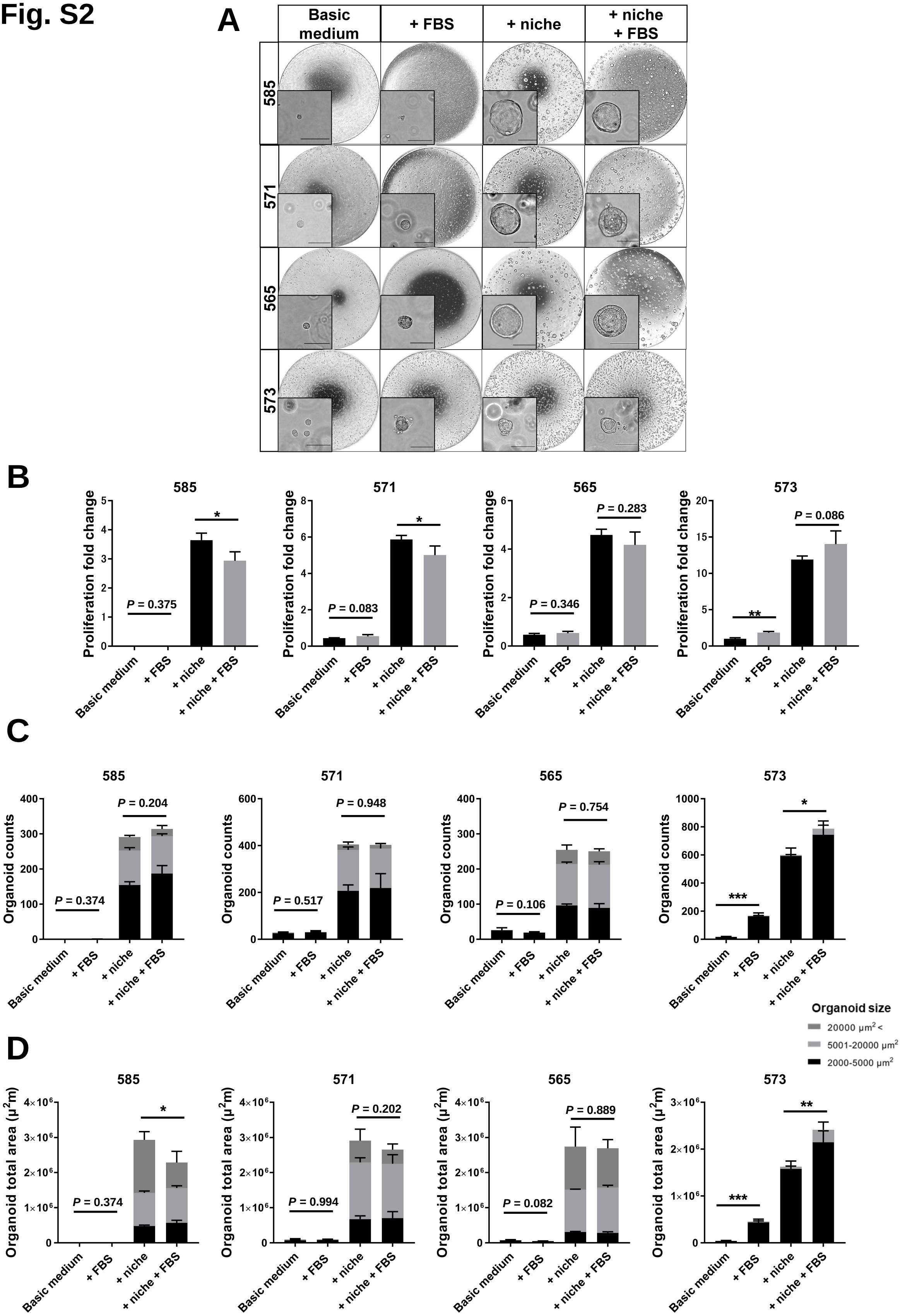

(continued on following page)

Fig. S2 *(continued)*

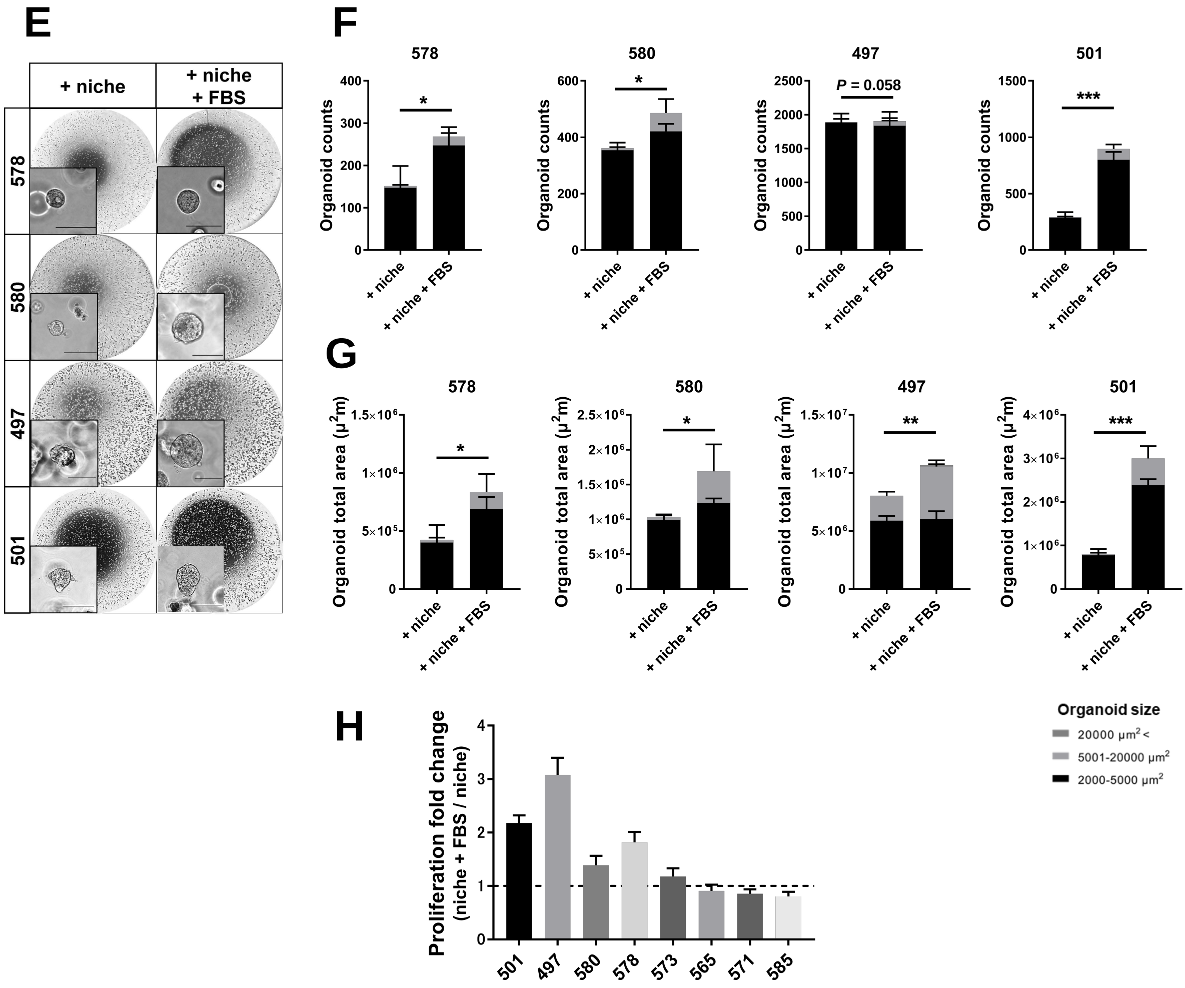

Fig. S2 The serum response of PDOs differed according to the tumor grade and Moffitt's classification. (A) Representative images of PDOs classified "Classical" subtype cultured in basic medium, serum medium, niche medium, or combined medium. Niche medium comprised the basic medium supplemented with the niche factors. Serum medium comprised the basic medium supplemented with only 5% fetal bovine serum (FBS). Combined medium comprised the basic medium supplemented with both niche factors and 5% FBS. Inset, highly magnified views show morphological features. Scale bars, 100 μm. (B) Quantification of the growth rate of "Classical" PDOs cultured the indicated medium. (C, D) Quantification of the number (C) and total area (D) of "Classical" PDOs cultured the indicated medium. (E) Representative images of PDOs classified "Basal-like" subtype cultured in niche medium, or combined medium. The three bars indicate organoid size. An area of 2000 μm² and more was identified as an organoid. (F, G) Quantification of the number (F) and total area (G) of "Basal-like" PDOs cultured the indicated medium. (H) Summary of serum response in all PDOs. The proliferation fold change in combined medium relative to niche medium was calculated (*P<0.05; **P<0.01; ***P<0.001). + FBS, serum medium; + niche, niche medium; + niche + FBS, combined medium.

Fig. S3

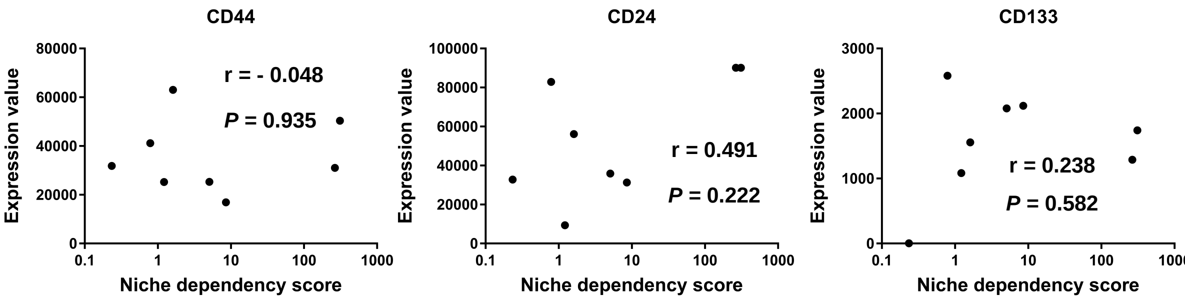

Fig. S3 Spearman's correlation analysis between the niche factor dependency and the expression levels of PDAC stem cell markers, including CD44, CD24, and CD133.

Fig. S4

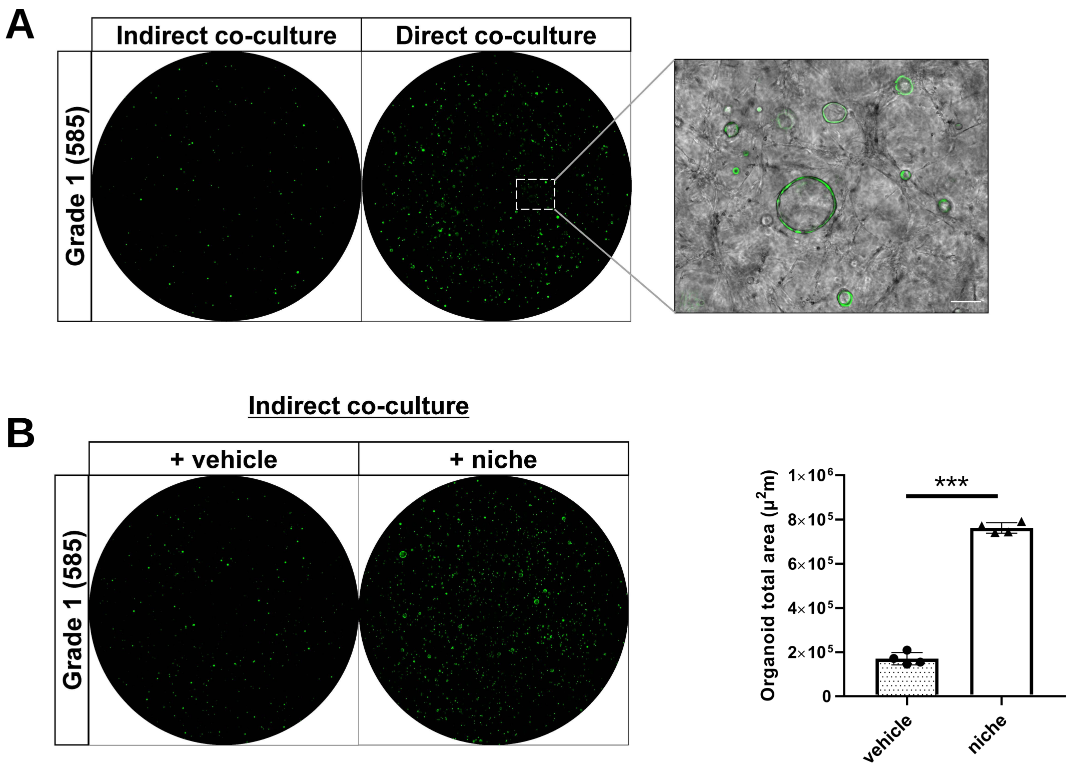

Fig. S4 Supplementation of niche factors to Grade1 PDOs following indirect co-culture with CAFs allowed organoid formations. (A) Representative fluorescence images (left) of Grade1 PDOs co-cultured indirectly or directly with CAF-1. High magnified fluorescence image (right) overlayed with phase-contrast image of Grade1 PDOs direct co-cultured with CAF-1. (B) Representative fluorescence images on day7 of Grade1 PDOs indirect co-cultured with CAF-1 in serum medium supplemented with vehicle or niche factors following indirect co-cultured for 10 days, and Quantification of the total area of PDOs (***P<0.001).

Fig. S5

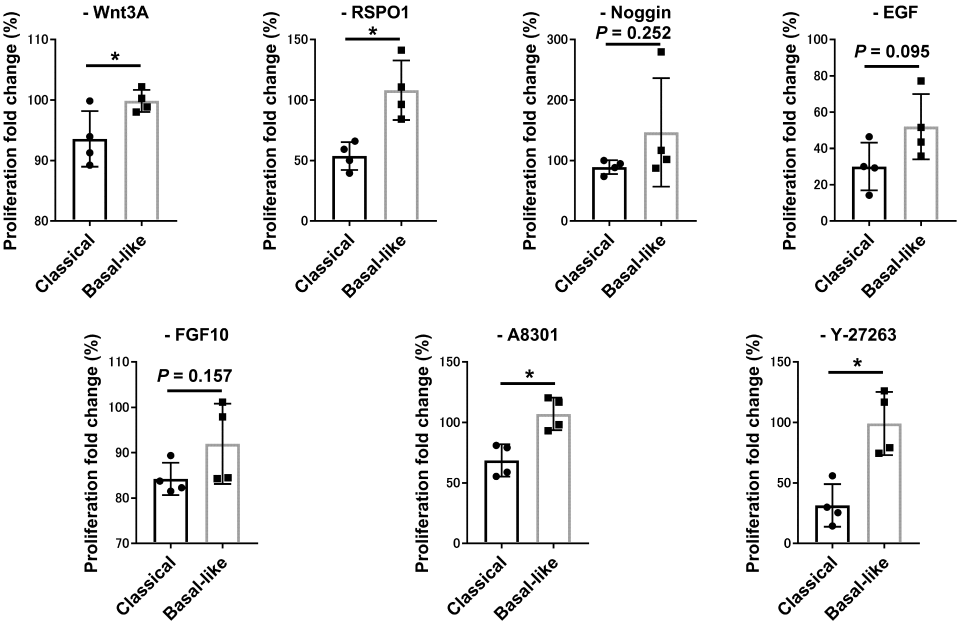

Fig. S5 The relationship between Moffitt's classification ("Classical" and "Basal-like") and the proliferation rate of PDOs in the absence of the indicated factors (**P<0.01; ***P<0.001).

Fig. S6

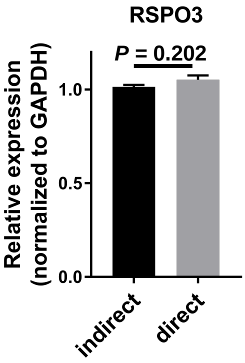

Fig. S6 qRT-PCR analysis of the mRNA expressions of RSPO3 in CAFs co-cultured indirectly or directly with PDO585. indirect, indirect co-culture; direct, direct co-culture.

Fig. S7

A

Organoid No.	501	497	580	578	573	565	571	585
Niche dependency scores (variables)	0.234	0.788	1.22	1.61	5.08	8.54	265	311

B

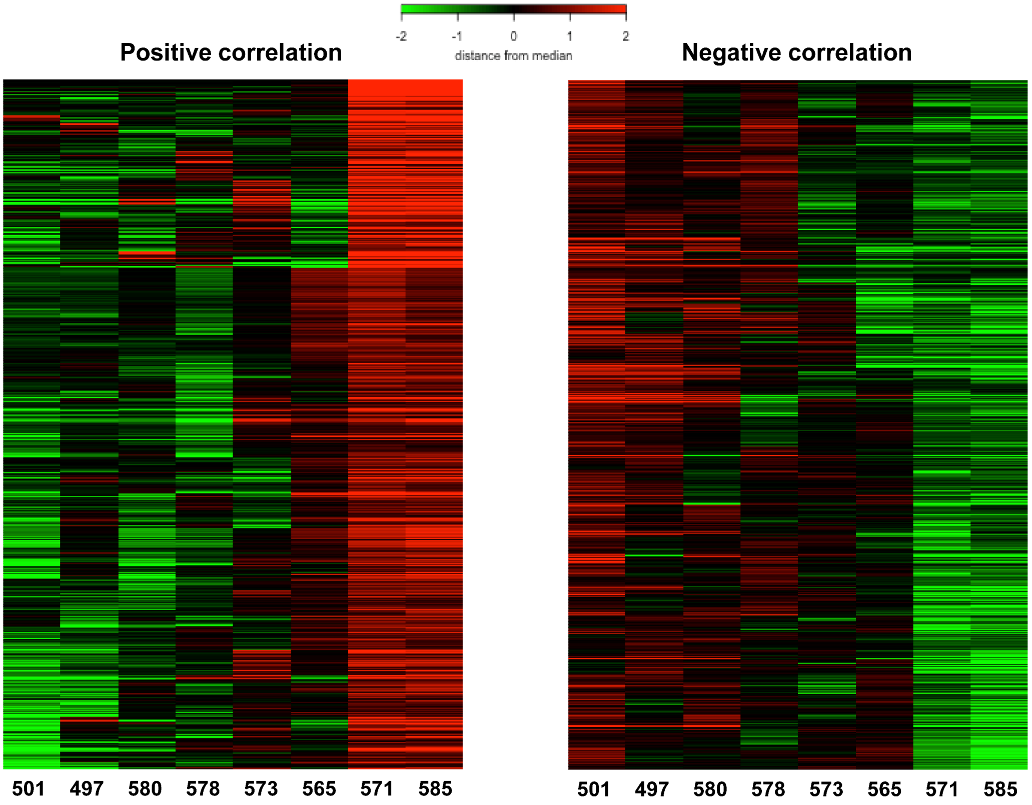

C

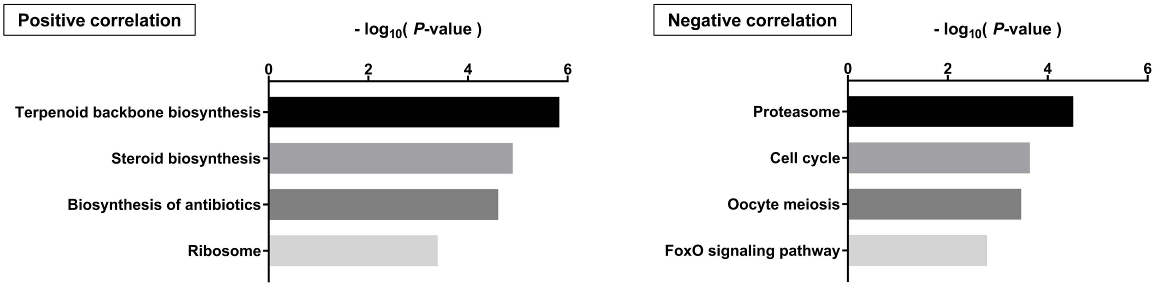

Fig. S7 Transcriptomic signatures induced by niche factors. **(A)** Niche dependency scores used as variables. **(B)** Heatmap of differentially expressed genes showing positive or negative correlation with niche factor dependency. A color key of Z score is shown. (C) KEGG pathway analysis of positive and negative correlation differentially expressed genes (FDR q-value < 0.1).
