# Supplementary material for: Subtypes in pancreatic ductal adenocarcinoma based on niche factor dependency show distinct drug treatment responses
Source: J Exp Clin Cancer Res. 2022 Mar 10;41:89. doi: 10.1186/s13046-022-02301-9 (PMC8908673; doi:10.1186/s13046-022-02301-9)
Supplement: Supplementary file 2 — Additional file 2: Table S1. Relationship betweentumor differentiation and clinicopathologic factors. [file 13046_2022_2301_MOESM2_ESM.docx]

**Table. S1**

|  |  | Tumor differentiation | | | |  |
| --- | --- | --- | --- | --- | --- | --- |
|  |  | well | | moderate / poor | |  |
|  |  | n=187 | (77%) | n=55 | (23%) | *P*  Value |
| Characterisitics |  |  |  |  |  |  |
| Age | <65 | 66 | (35%) | 18 | (33%) | 0.725 |
|  | ≧65 | 121 | (65%) | 37 | (67%) |  |
| pT category | pT1/pT2 | 25 | (13%) | 7 | (13%) | 0.902 |
|  | pT3/pT4 | 162 | (87%) | 48 | (87%) |  |
| Lymph node metastasis | No | 73 | (39%) | 21 | (38%) | 0.909 |
|  | Yes | 114 | (61%) | 34 | (62%) |  |
| UICC stage | IA/IB | 16 | (9%) | 6 | (11%) | 0.383 |
|  | IIA/IIB | 154 | (82%) | 47 | (85%) |  |
|  | III/IV | 17 | (9%) | 2 | (4%) |  |
| Perilymphatic invasion | No | 120 | (64%) | 35 | (64%) | 0.942 |
|  | Yes | 67 | (36%) | 20 | (36%) |  |
| Perivascular invasion | No | 112 | (60%) | 26 | (47%) | 0.097 |
|  | Yes | 75 | (40%) | 29 | (53%) |  |
| Perineural invasion | No | 40 | (21%) | 13 | (24%) | 0.723 |
|  | Yes | 147 | (79%) | 42 | (76%) |  |
| Pathological margin | Negative | 166 | (89%) | 49 | (89%) | 0.947 |
|  | Positive | 21 | (11%) | 6 | (11%) |  |
| Neoadjuvant chemotherapy | Yes | 44 | (24%) | 11 | (20%) | 0.583 |
|  | No | 143 | (76%) | 44 | (80%) |  |
| Adjuvant chemotherapy | Yes | 161 | (86%) | 47 | (85%) | 0.650 |
|  | No | 25 | (13%) | 7 | (13%) |  |
|  | Unknown | 1 | (1%) | 1 | (2%) |  |
| UICC, Union for International Cancer Control | | |  |  |  |  |

**Supplementary Table Legends:**

**Relationship between tumor differentiation and clinicopathologic factors**

The χ^2^ test was performed to analyze correlations between tumor differentiation and the observed clinicopathologic characteristics.
